# Hoarding disorder: evidence and best practice in primary care

**DOI:** 10.3399/bjgp23X732513

**Published:** 2023-03-31

**Authors:** Sharon Morein-Zamir, Sanjiv Ahluwalia

**Affiliations:** School of Psychology and Sports Science, Anglia Ruskin University, Cambridge.; School of Medicine, Anglia Ruskin University, Chelmsford.

## INTRODUCTION

With clinical, social, environmental, and legislative considerations, hoarding disorder (HD) poses unique challenges in relation to its diagnosis and treatment. Hoarding is characterised by excessive clutter and difficulty discarding. While many individuals may report dissatisfaction and difficulties with such symptoms alongside excessive acquisition, only when these lead to clinically significant distress and/or impairment in social, occupational, or other important areas of functioning is the diagnosis of HD considered.^1^ Hoarding is associated with significant physical, psychological, and social morbidity leading to reduced quality of life. Even safety can be affected by possessions that congest and clutter active living areas and substantially compromise their intended use. Relationships within the household, with extended family and friends, and even with neighbours may come under stain. HD is found across the world, with a prevalence of approximately 2% and with similar rates for males and females.^2^ Despite the prevalence and considerable personal costs, recognising and providing appropriate care can prove challenging. Only in 2013 was HD listed in the *Diagnostic and Statistical Manual of Mental Disorders* (DSM-5)^1^ and in 2019 was it formally listed in the International Classification of Diseases (ICD-11, code F42.6B24).^3^ Beforehand, HD was classified under obsessive compulsive disorders (OCD). However, most patients with HD notably do not display the cardinal OCD symptoms of obsessions and compulsions, with further evidence of differing onset, course, pathophysiology, and treatment responses.^1^ This classification history has hindered research, clinical understanding, and treatment development.

## SYMPTOMS AND DIAGNOSIS

In clinical settings, recognising hoarding symptoms can prove challenging for several reasons. Hoarding behaviours often manifest by early adulthood, but patients and their families typically do not disclose difficulties to health professionals or seek help until decades later, if at all. This is due in part to the condition’s insidious nature together with management of symptom severity by family and carers. However, the key reason health professionals may not find out about hoarding-related difficulties is limited insight. Insight is often poor or absent, whereby the individual is convinced their hoarding-related beliefs and behaviours are not problematic, despite evidence to the contrary. Though insight serves as a specifier in both the DSM-5 and ICD-11 to further clarify the nature of HD, the frequency and severity of poor insight have yet to be explored. Thus, patients will not seek help, nor disclose their difficulties when asked, and may not acknowledge them when explicitly discussed. Moreover, traumatic life events and hardship are frequent in those with HD^4^ and can lead to social vulnerability, isolation, distrust or fear of engaging with service providers, and housing insecurity. Further, societal stigma and feelings of shame, exacerbated by depictions in popular media, reduce disclosure even in those with reasonable or good insight.^5^

Hoarding-related difficulties often come to the forefront when patients seek support and treatment for other mental health or physical conditions. Hoarding can also emerge as a barrier to other treatments given concerns about hygiene, safety, or access to the home. Depression is the most common comorbidity, linked to the high frequency of adverse life events often characterised by trauma, grief, and loss.^4^^,^^6^ Other common comorbidities include OCD (18%) and attention deficit hyperactivity disorder, particularly inattentive type (up to 28%).^7^ Diagnosis should consider whether hoarding is secondary to other health problems including mental health conditions, such as schizophrenia, dementia, or physical ailments that limit mobility or the patient’s ability to maintain their home environment. A strong attachment to possessions constituting the hoard with fear of them being discarded, lost, or forcefully taken away would suggest HD *per se*. When there is no difficulty discarding, a diagnosis of HD would not be appropriate. During consultation, if GPs suspect hoarding they can ask about symptoms in an equanimous and even indirect manner, and if needed arrange a home visit. There are also available tools online, such as the clutter image rating scale.^8^ Greater awareness of HD can not only aid the individual and family in support for the condition but also when interacting with service providers in health care and further afield.

## TREATMENT AND SUPPORT

Once hoarding-related issues have been identified and assessed, a long-term coordinated supportive approach can help patients and their families. In the UK, local care services play a significant role for those who hoard. Preliminary evidence indicates that, although comprising a small population, individuals who hoard pose considerable economic burden to services^9^ including local authorities. Given the diversity of first-line service providers that encounter people who hoard, local authorities have recognised the need for adopting coordinated multi-agency partnerships.^10^ These partnerships include not only NHS Trusts and district councils, but also housing associations, safeguarding adult boards, mental health charities, and ambulance, fire, and police services. Though many local authorities have hoarding multi-agency protocols, their implementation and effectiveness has been variable as is the training to service providers regarding hoarding. The UK Care Act (2014) set out the legal framework for safeguarding adults, providing considerable responsibilities to local authorities. The act considers ‘self-neglect’ within the safeguarding arrangements and explicitly refers to hoarding as an example, though the threshold is relatively high. With the UK Health and Care Act (2022), Integrated Health Boards are set to provide additional structure and require even greater health and social care collaboration. As healthcare professionals with expertise in physical health, mental health, and continuity of care expertise, GPs and other healthcare professionals in the surgery can play a vital role in fostering good communication and coordination with other frontline professionals. Moreover, GPs can enhance patient care by strategically utilising local protocols, key contacts, and resources available to them and their teams.

GPs may not always be the first port of call but once involved can raise concerns and awareness of problems, including psychoeducation building on freely available resources (for example, charity websites such as MIND). Cultivating trust and providing compassion is often essential, requiring a long-term relationship. Recognising HD as an ongoing and chronic mental health condition can help avoid judgement and facilitate engagement from all involved. At times, clearing out possessions can be suggested as a solution. A patient with no HD may even welcome such an intervention. Importantly, for patients with HD, clearances can worsen their mental health, cause significant distress, and only provide a temporary intervention. Hence, they are to be avoided whenever possible and, if there is no other recourse, need to be discussed in advance and conducted with the consent and participation of the individual. National Institute for Health and Care Excellence guidelines recommend cognitive behavioural therapy and selective serotonin reuptake inhibitor treatment for HD, though there is insufficient evidence for their effectiveness. This is in part because good-quality treatment trials are missing due to the heterogeneity of samples and, until recently, no clear diagnostic criteria. There are no HD-specific specialist services, and support can be variable across the NHS. Psychological and psychiatric research to date has focused on those with comorbid OCD or those with reasonable insight so may not generalise to all patients seen by GPs.^11^ With informed experience about capacity and choice^12^ primary care is integral to issues regarding safeguarding adults. This is especially important when there are safety concerns to the patient, others in the home, and in some cases those living in close proximity, such as neighbours. With the chronic nature of HD and at times its seeming intractable nature, harm reduction rather than treatment may be a more feasible approach, considering risk management concerns.

**Box 1.** Key points about caring for those with hoarding disorder

Hoarding disorder (HD) only exists as a psychiatric condition with clear diagnostic criteria since 2013, hampering research and treatment. Symptoms include difficulty discarding possessions and their excessive accumulation, along with clutter of active living areas to a degree that compromises their intended use. Individuals with HD are often characterised by limited insight, which can be exacerbated by stigma, with both substantially inhibiting disclosure and help seeking to healthcare professionals. Living conditions can directly impact not only patient wellbeing and safety but also others in the home, visitors, and at times also those living in close physical proximity. A diverse set of front-line providers can often be the first to encounter individuals with HD (for example, housing officers, environmental health, fire, police, and ambulance services), but may not have a long-term relationship with the individual or appropriate clinical training. Current best practice entails close and coordinated engagement with all relevant agencies to facilitate long-term patient-centred support and care.

## CONCLUSION

In conclusion, difficulties related to hoarding are more pervasive than previously thought, with HD being associated with considerable psychological, physical, and social harms. GPs and their teams can lead in the identification and long-term care of these patients alongside a coordinated engagement with a wide variety of existing support services. Future research may further help to develop and evaluate screening tools for hoarding in primary care settings as well as how GPs can best engage with patients, family members, and carers.
